# Molecular Signatures Related to the Virulence of Bacillus cereus Sensu Lato, a Leading Cause of Devastating Endophthalmitis

**DOI:** 10.1128/mSystems.00745-19

**Published:** 2019-12-03

**Authors:** Jian Yuan, Yu-Yu Li, Yi Xu, Bian-Jin Sun, Jiao Shao, Die Zhang, Kai Li, Dan-Dan Fan, Zheng-Bo Xue, Wei-Hua Chen, Clara Pak, Yong-Liang Lou, Jian-Zhong Su, Mei-Qin Zheng

**Affiliations:** a School of Ophthalmology and Optometry and Eye Hospital, School of Biomedical Engineering, Wenzhou Medical University, Wenzhou, China; b Wenzhou Institute, University of Chinese Academy of Sciences, Wenzhou, China; c Zhejiang Provincial Key Laboratory for Technology and Application of Model Organisms, Key Laboratory of Laboratory Medicine, Ministry of Education, China, School of Laboratory Medicine and Life Sciences, Wenzhou Medical University, Wenzhou, China; d Key Laboratory of Molecular Biophysics of the Ministry of Education, Hubei Key Laboratory of Bioinformatics and Molecular Imaging, Department of Bioinformatics and Systems Biology, College of Life Science and Technology, Huazhong University of Science and Technology, Wuhan, Hubei, China; e College of Life Science, Henan Normal University, Xinxiang, Henan, China; f Huazhong University of Science and Technology Ezhou Industrial Technology Research Institute, Ezhou, Hubei, China; g University of Rochester Medical Center, Rochester, New York, USA; Institute for Systems Biology

**Keywords:** endophthalmitis, *Bacillus*, pangenome, virulence, evolution

## Abstract

*Bacillus* endophthalmitis is a devastating eye infection that causes rapid blindness through extracellular tissue-destructive exotoxins. Despite its importance, knowledge of the phylogenetic relationships and population structure of intraocular *Bacillus* spp. is lacking. In this study, we sequenced the whole genomes of eight *Bacillus* intraocular pathogens independently isolated from 8/52 patients with posttraumatic *Bacillus* endophthalmitis infections in the Eye Hospital of Wenzhou Medical University between January 2010 and December 2018. Phylogenetic analysis revealed that the pathogenic intraocular isolates belonged to Bacillus cereus, Bacillus thuringiensis and Bacillus toyonensis. To determine the virulence of the ocular isolates, three representative strains were injected into mouse models, and severe endophthalmitis leading to blindness was observed. Through incorporating publicly available genomes for *Bacillus* spp., we found that the intraocular pathogens could be isolated independently but displayed a similar genetic context. In addition, our data provide genome-wide support for intraocular and gastrointestinal sources of *Bacillus* spp. belonging to different lineages. Importantly, we identified five molecular signatures of virulence and motility genes associated with intraocular infection, namely, *plcA-2*, *InhA-3*, *InhA-4, hblA-5*, and *fliD* using pangenome-wide association studies. The characterization of overrepresented genes in the intraocular isolates holds value to predict bacterial evolution and for the design of future intervention strategies in patients with endophthalmitis.

**IMPORTANCE** In this study, we provided a detailed and comprehensive clinicopathological and pathogenic report of *Bacillus* endophthalmitis over the 8 years of the study period. We first reported the whole-genome sequence of *Bacillus* spp. causing devastating endophthalmitis and found that Bacillus toyonensis is able to cause endophthalmitis. Finally, we revealed significant endophthalmitis-associated virulence genes involved in hemolysis, immunity inhibition, and pathogenesis. Overall, as more sequencing data sets become available, these data will facilitate comparative research and will reveal the emergence of pathogenic “ocular bacteria.”

## Abstract (precis)

In this study, we provided a detailed and comprehensive clinicopathological and pathogenic report of *Bacillus* endophthalmitis over the 8 years of the study period. We first reported the whole-genome sequence of *Bacillus* spp. causing devastating endophthalmitis and found that Bacillus toyonensis is able to cause endophthalmitis. Finally, we revealed significant endophthalmitis-associated virulence genes involved in hemolysis, immunity inhibition, and pathogenesis. Overall, as more sequencing data sets become available, these data will facilitate comparative research and will reveal the emergence of pathogenic “ocular bacteria.”

## INTRODUCTION

Endophthalmitis is an ocular inflammatory condition within the anterior or posterior segment of the eye, which is commonly caused by bacterial or fungal infections. Despite aggressive treatment and surgical intervention, endophthalmitis frequently leads to partial or complete loss of vision. Posttraumatic endophthalmitis results from bacterial infections following ophthalmic surgery and trauma. One of the most explosive and devastating forms of posttraumatic endophthalmitis is caused by Bacillus cereus, which typically migrates throughout the eye causing a rapid and severe intraocular inflammatory response, leading to a loss of functional vision. Although posttraumatic endophthalmitis caused by B. cereus is rare, it is ranked second behind that caused by staphylococci, occurring in ∼2 to 7% of all penetrating eye wounds (1).

Bacillus cereus is a Gram-positive, endospore-forming rod that is ubiquitous in the environment under aerobic-to-facultative conditions and bears a close genetic and phenotypic relationship to several other “Bacillus cereus group” species, including B. thuringiensis, B. anthracis, B. mycoides, B. pseudomycoides, B. weihenstephanensis, B. cytotoxicus, and B. toyonensis (2, 3). B. cereus endophthalmitis is a devastating eye infection that causes blindness due to the release of a variety of extracellular tissue-destructive exotoxins (4, 5), including phosphatidylinositol-specific phospholipase Cs (PI-PLCs) (6), immune inhibitor A metallopeptidase (InhA) (7), and hemolysin BL (HBL) (8).

A molecular understanding of the genetic basis and evolution of pathogens for host adaptation is important for the control of infection and therapeutic intervention. Advances in genome sequencing have facilitated the comparison of genomes from large numbers of bacterial isolates and revealed how the pathogenicity of bacterial clones can evolve via mutation and horizontal gene transfer (9, 10). From a bacterial perspective, the acquisition of new genes provides the flexibility to adapt and take advantage of novel niches. Although an array of studies have provided insight into genetically and phenotypically diverse environmental *Bacillus* species strains and clinical isolates associated with gastrointestinal and ocular infections (2, 6, 11–14), genomic information and the key determinants of virulence leading to posttraumatic endophthalmitis *in vivo* remain unclear (15, 16).

In this study, we present a retrospective analysis of 52 cases of posttraumatic *Bacillus* endophthalmitis between January 2010 and December 2018 and determine the pathogenicity of intraocular *Bacillus* species isolates in mouse models of bacterial endophthalmitis. To investigate the genomic characteristics and molecular signatures of intraocular strains, we sequenced the genomes of eight *Bacillus* intraocular isolates spanning different patients. Incorporating publicly available genome sequences for *Bacillus* spp., we investigated the population structure and evolution of intraocular isolates through phylogenomic analysis. We also performed pangenome-wide association studies to identify the association between gene pools and disease outcomes and to identify different sets of accessory genes associated with virulence traits in endophthalmitis. These data have important implications for our understanding of the pathogenicity in *Bacillus* spp. and will facilitate future epidemiology and therapeutic studies.

## RESULTS

### Clinicopathological characteristics of *Bacillus* endophthalmitis.

We followed 52 patients with posttraumatic endophthalmitis attending the Affiliated Eye Hospital of Wenzhou Medical University, China, between January 2010 and December 2018. The patient inclusion criteria were as previously described (15), namely, infective endophthalmitis caused by penetrating wounds, positive Gram stains for vitreous biopsy specimens or aqueous humor positivity, *Bacillus* spp. that grew on the vitreous or aqueous humor, and absence of other infectious diseases. Finally, 52 cases (48 males and 4 females) with a mean age of 47.3 ± 17.3 years (range: 3 to 82 years) were included in this retrospective study (Table 1). The follow-up time was 20.2 ± 9.5 months (range: 8 to 38 months). Penetrating trauma among the patients was caused by iron (*n* = 32), pebbles (*n* = 10), branches (*n* = 3), electrical wires (*n* = 2), firecrackers (*n* = 2), glass (*n* = 1), and other reasons (*n* = 2). Of the patients, 92% (48/52) developed rapid and aggressive ocular pain, redness, a thick yellowish discharge, and notably reduced visual acuity. Despite aggressive therapeutic and surgical intervention, endophthalmitis generally resulted in a partial or complete loss of vision. The visual acuity of the 52 patients was as follows: 55% (29/52) with a complete loss of vision, including no light perception (NLP) (14/29) and enucleation of the eye (15/29); 17% (9/52) with visual acuity of only light perception (LP); 15% (8/52) with hand movements (HM); 4% (2/52) with visual acuity of the count finger (CF); and 8% (4/52) with limited visual acuity ranging from 20/200 to 20/40.

**TABLE 1:** Patient information

Variable (*n* = 52)	Value
Demographic data	
Age (yr), mean ± SD (range)	47.3 ± 17.3 (3–82)
Sex (no. male/no. female)	48/4
Occupation (*n*)	
Worker	31
Farmer	17
Student	4
Baseline characteristic (*n*)	
Laterality (left/right)	30/22
Character of foreign body	
Iron	32
Pebbles	10
Branch	3
Electrical wire	2
Firecracker	2
Glass	1
Unclear	2
Size of wound (mm^2^), mean ± SD (range)	4.6 ± 2.4 (1–12)
Onset time (h), mean ± SD (range)	20.1 ± 26.3 (2–168)
Follow-up time (mo), mean ± SD (range)	20.2 ± 9.5 (8–38)
Treatment (*n*)	
Enucleation	15
Vitrectomy	33
Removal of the foreign body	4
Final visual acuity (*n*)	
No light perception	29
Light perception	9
Hand movement	8
Count finger	2
Other	4

**FIG 1:**
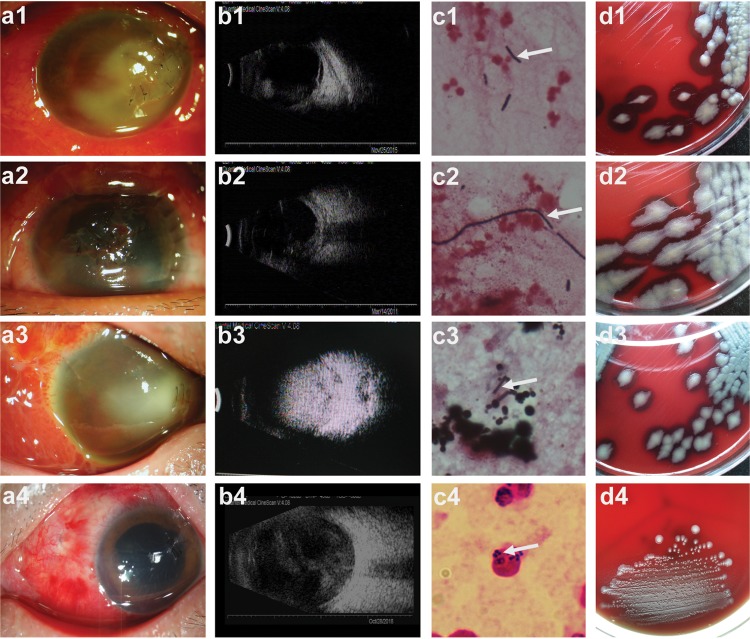
Clinicopathological features of posttraumatic endophthalmitis. Anterior segment photograph (a1 to a4, ×10), B scan ultrasound images (b1 to b4), Gram stain picture (c1 to c4, ×1,000), and colony morphology (d1 to d4) showing that patients with posttraumatic endophthalmitis were infected by *Bacillus* spp. (patients 1 to 3) and Staphylococcus epidermidis (patient 4). In the Gram stain pictures (c1 to c4), white arrows point to a bipolarly stained *Bacillus* species and Staphylococcus epidermidis.

Figure 1 shows the clinicopathological characteristics of three representative posttraumatic endophthalmitis cases (Fig. 1, rows 1 to 3) and *Staphylococcus* endophthalmitis controls (Fig. 1, row 4). The typical clinical manifestations were severe swelling of the eyelids, conjunctival congestion, conjunctival chemosis, corneal edema, and hypopyon in the anterior chamber (Fig. 1, a1 to a3, anterior segment photograph). The shape, brightness, position, and after-movement of echogenic dots in the dark area of vitreous body are shown (Fig. 1, b1 to b3, B scan ultrasound images). The vitreous smear indicated a Gram-positive bacterium as the causative factor (Fig. 1, c1 to c3). The bacteria were often arranged in pairs or chains and were typically located externally to phagocytes. Upon 24 h of vitreous humor and aqueous humor culture in sheep blood agar medium, the strains formed large gray-white opaque colonies of different sizes, irregular edges, wax-like clusters, and obvious hemolytic rings (Fig. 1, d1 to d3). Colony morphology combined with biochemical assessments (API 20E and API 50CHB strip identification) showed that the strains belonged to the Bacillus cereus group. Compared to *Bacillus* infection, Staphylococcus epidermidis, which is the more prevalent pathogen in posttraumatic endophthalmitis (17), caused fewer inflammatory or pathogenic effects (Fig. 1, a4 to d4). At the same time, the vitreous smear of the patients indicated that S. epidermidis can be easily phagocytized by neutrophils, which differs from Bacillus cereus (Fig. 1, c1 to c4).

### Taxonomic status and pathogenicity of the intraocular isolates.

To determine the taxonomic status of the eight intraocular isolates, we sequenced the whole genomes of the eight intraocular strains LY1, LY2, LY5, LY6, LY7, LY178, LY557, and LY460 (see Table S1 in the supplemental material) and performed phylogenetic analysis using RAxML (18). As shown in Fig. S1, although all intraocular isolates were members of the B. cereus group, they belonged to independent species. *Bacillus* strains LY1, LY5, LY6, and LY178 were members of B. thuringiensis; *Bacillus* strains LY2, LY7, and LY460 were B. cereus sensu stricto; and *Bacillus* strain LY557 is a novel strain of *B. toyonensis* that is the first to be reported in endophthalmitis.

10.1128/mSystems.00745-19.1 FIG S1 Phylogenetic tree of *Bacillus* spp. and position of the intraocular strains. Maximum likelihood phylogenetic tree of 135 *Bacillus* species isolates inferred from 192,201 aligned nucleotide characters in the 1,184 core genes. Scale bars indicate the average number of substitutions per site. Intraocular and gastrointestinal isolates in this study are colored red. Download FIG S1, JPG file, 2.2 MB. Copyright © 2019 Yuan et al. 2019 Yuan et al. This content is distributed under the terms of the Creative Commons Attribution 4.0 International license.

10.1128/mSystems.00745-19.6 TABLE S1 Genome statistics of sequenced *Bacillus* species strains. Download Table S1, XLSX file, 0.01 MB. Copyright © 2019 Yuan et al. 2019 Yuan et al. This content is distributed under the terms of the Creative Commons Attribution 4.0 International license.

**FIG 2:**
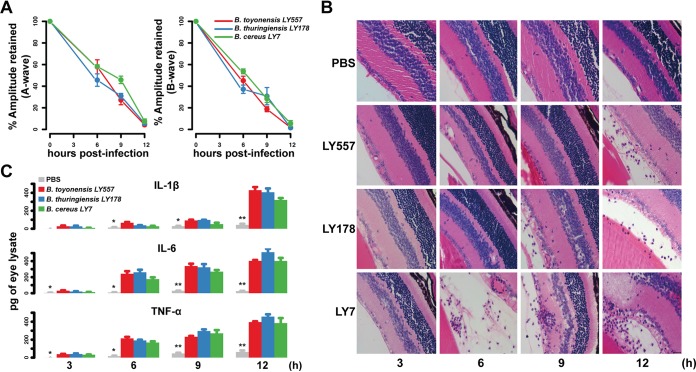
Pathogenicity of the intraocular *Bacillus* strains LY557, LY178, and LY7 in a mouse model of bacterial endophthalmitis. (A) Electroretinogram responses were recorded at indicated time points, and the percent amplitudes of A- and B-waves retained in infected eyes were compared to that of PBS-injected controls and presented as mean ± SD ERG amplitudes. (B) Histopathological analysis of the posterior segment was performed to assess pathogen-induced inflammation (×100). (C) ELISA was used to quantify the expression of proinflammatory cytokines at indicated time points. (*, *P* < 0.05; **, *P* < 0.01; Student’s *t* test, PBS versus infected).

To investigate whether the *Bacillus* isolates caused endophthalmitis, we injected wild-type C57BL/6 mice with the three representative isolates of *Bacillus* species and phosphate-buffered saline (PBS) controls and analyzed retinal function, histology, and inflammatory mediator expression of the vitreous chamber *in vivo* at 3 h, 6 h, 9 h, and 12 h postinjection as previously described (19). We measured scotopic electroretinography (ERG) responses to determine whether infection with the isolates influenced retinal function. We found that both A (57%)- and B (45%)-wave amplitudes were significantly reduced in infected eyes 6 h postinfection (hpi), (*P *= 6.3 × 10^−7^ and 7.7 × 10^−8^ for A-wave and B-wave, respectively, Student’s *t* test) and almost completely disappeared at 12 hpi (Fig. 2A). To further elucidate the degree of retinal damage caused by *Bacillus* pathogens, we infected mice with normal ocular surface flora of Staphylococcus epidermidis and performed electroretinogram experiments. We found that *Bacillus* was able to cause more severe retinal damage after 6 h of infection. These results were consistent with the clinical phenotype (Fig. S2A). Compared to untreated eyes (Fig. S2B), hematoxylin and eosin (HE) staining in injected mouse eyeball tissue revealed a large number of inflammatory cells in the vitreous cavity following infection (6 hpi in strain LY7, 9 hpi in strain LY178, and 12 hpi in strain LY557) (*P < *0.01, Student’s *t* test [Fig. 2B and Fig. S2C]). In addition, the expression of IL-1β in eyeball homogenates rapidly increased at 12 hpi, while that of IL-6 and TNF-α increased dramatically at 9 hpi (*P < *0.01, Student’s *t* test [Fig. 2C]). Together, these results validate that the intraocular isolates of *Bacillus* spp. directly caused severe intraocular inflammation and retinal damage leading to vision loss. Importantly, these results indicate that *B. toyonensis* LY557 is an emerging but rare ocular pathogen capable of severe intraocular inflammation and retinal damage.

10.1128/mSystems.00745-19.2 FIG S2 Histology and inflammatory response of the vitreous chamber. (A) Electroretinogram responses of indicated pathogen infections were recorded at 6-h time points and the percent amplitude of A- and B-waves retained in infected eyes was compared to that of PBS-injected controls and presented as mean ± SD ERG amplitudes. (B) HE staining of untreated eyeball tissues. (C) Number of inflammatory cells in the vitreous cavity after infection. Download FIG S2, JPG file, 1.6 MB. Copyright © 2019 Yuan et al. 2019 Yuan et al. This content is distributed under the terms of the Creative Commons Attribution 4.0 International license.

### Phylogenetic structure and evolution of intraocular strains.

**FIG 3:**
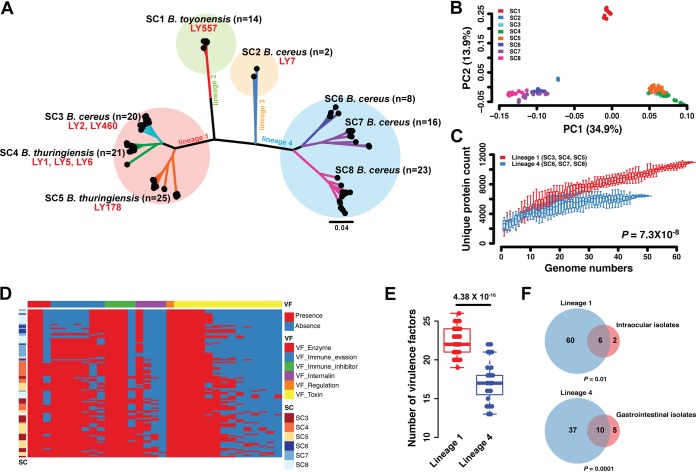
Phylogeny and pangenome of *Bacillus* spp. (A) Phylogeny of 129 intraocular isolate-related genome sequences based on core gene SNPs. Each subcluster (SC) with more than one genome is highlighted in alternating colors by RAMI and labeled with representative species and number of isolates. (B) PCA based on the presence of common (5 to 95% prevalence) accessory genes. (C) Accumulation curves for the total number of unique genes given a number of genomes analyzed for different strains of lineage 1 (in red) and lineage 4 (in blue). The vertical bars correspond to standard deviations after repeating 100 random input orders of the genomes. *P* values were calculated using Wilcoxon rank sum tests. (D) Heatmaps indicate the presence (red) or absence (blue) of virulence genes across the 6 subclusters. (E) Box plot of virulence gene numbers of lineage 1 and lineage 4. *P* values were calculated using Wilcoxon rank sum tests. (F) Bacteria isolated from intraocular tissue significantly enriched in lineage 1 and gastrointestinal isolates significantly enriched in lineage 4; hypergeometric test.

A maximum likelihood (ML) tree of 129 intraocular isolate-related genome sequences was created based on 192,201 core genome single nucleotide polymorphisms (SNPs) in the 1,184 core genes (Fig. 3A). Phylogenetic analysis showed a wide diversity of clearly defined lineages within the genus. We divided 129 genomes into 4 lineages, including 8 subclusters (SC1 to -8) based on the analysis of the core ML tree using RAMI (20) (Fig. 3A). Principal-component analysis (PCA) using 5,368 accessory genes of intermediate frequency (5% to 95%) clearly distinguished the four groups (Fig. 3B), which were comparable to phylogenetic lineages. These data sets provide whole-genome support for the theory that the phylogroups constitute discrete bacterial populations that are evolving independently. As shown in Fig. 3A, although our eight intraocular isolates were distributed in lineages 1, 2, and 3, they were mainly comprised (6/8) of lineage 1. Using a pangenome approach, the *Bacillus* population had an open pangenome, indicating a far larger gene pool within the *Bacillus* population and frequent gene gain and loss events (Fig. 3C). When comparing the total number of unique genes to a given number of genomes, we found that lineage 1, which included intraocular isolates, had more unique gene numbers than lineage 4, another dominant lineage of the *Bacillus* genus (*P = *7.3 × 10^−8^, Wilcoxon rank sum test).

The analysis of virulence genes has formed a core narrative in our understanding of pathogen evolution. Several genetic loci have been identified as virulent based on the virulence factor database (VFDB) (21), which includes gene clusters involved in enzyme activity, immune evasion, immune inhibition, and toxins. Although these virulence determinants have been characterized in B. cereus ATCC 10987 and ATCC 14579 genomes, their distribution across members of the genus remains unclear. From the clustering distribution of pathogenic genes (Fig. 3D), it is evident that the virulence genes form variable patterns between lineage 1 and lineage 4. Analysis of the number of virulence factors between the two lineages indicated a significantly higher prevalence of virulence genes in lineage 1 (*P = *4.38 × 10^−16^, Wilcoxon rank sum test [Fig. 3E]), which implied that this lineage has higher pathogenicity than lineage 4. In addition, we sequenced the whole genomes of two gastrointestinal isolates and collected 13 genomes of publicly available gastrointestinal strains (Fig. S1). We found that the intraocular strains were enriched in lineage 1, in which isolates showed more plasticity and pathogenicity, while gastrointestinal isolates were enriched in lineage 4 (*P < *0.01, hypergeometric test [Fig. 3F]).

### Molecular signatures of pathogenicity with intraocular pathogens.

**FIG 4:**
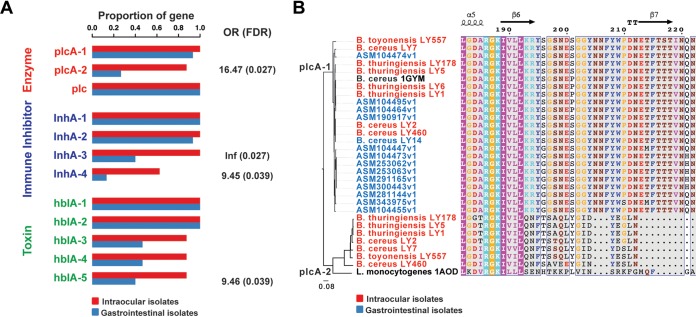
Virulence genes associated with intraocular pathogens. (A) Frequency of virulence genes among bacteria isolated from different human sources. OR, odds ratio for association between the presence of the genes and intraocular versus gastrointestinal infection; *P* values were calculated using Fisher’s exact test. (B) Neighbor-joining (NJ) tree and multiple alignment of *plcA-1* and *plcA-2* protein sequences across the known B. cereus (PDB accession no. 1GYM), L. monocytogenes (PDB accession no. 1AOD), and intraocular isolates.

To investigate the genetic determinants of intraocular pathogens, we identified four key virulence gene loci (*plcA-2*, *inhA-3*, *inhA-4*, and *hblA-5*) that were significantly associated with intraocular infection (*P < *0.01, one-sided Fisher’s exact test [Fig. 4A]). Notably, *plcA-2* showed the strongest association, achieving pangenome-wide significance after correction for multiple testing. Furthermore, we found that the intraocular pathogen-associated *plcA-2* gene is more comparable to Listeria monocytogenes *plcA* (PDB accession no. 1AOD [22]) than canonical *Bacillus plcA* (PDB accession no. 1GYM [23]), which encodes a secreted phosphatidylinositol-specific phospholipase C (PI-PLC) by BLASTP (Fig. 4B and Fig. S3) and homology modeling (Fig. S4). In addition, we found that intraocular *Bacillus plcA-2* and Listeria monocytogenes *plcA* both lack the Vb β-strand (Fig. 4B), which may lead to weaker cleavage activity on glycosylphosphatidylinositol (GPI)-anchored proteins and increased virulence (24). To extend these observations, we performed pangenome-wide association studies (PGWAS) using Scoary (25), screening 19,985 genes between intraocular and gastrointestinal isolates. The most significant pangenome-wide association was the flagellar cap protein FliD gene (false-discovery rate [FDR] = 0.024, Bonferroni correction [Table S2]).

10.1128/mSystems.00745-19.3 FIG S3 Multiple alignment of *plcA* protein sequences. Download FIG S3, PDF file, 0.1 MB. Copyright © 2019 Yuan et al. 2019 Yuan et al. This content is distributed under the terms of the Creative Commons Attribution 4.0 International license.

10.1128/mSystems.00745-19.4 FIG S4 *Bacillus* PI-PLC molecular model. Homology modeling analysis has revealed that a small β-strand (Vb) is present in B. cereus *plcA* and *B. toyonensis* strain LY557 *plcA-1* but is absent in the enzyme from L. monocytogenes *plcA-2* and *B. toyonensis* strain LY557 *plcA-2*. Download FIG S4, JPG file, 2.3 MB. Copyright © 2019 Yuan et al. 2019 Yuan et al. This content is distributed under the terms of the Creative Commons Attribution 4.0 International license.

10.1128/mSystems.00745-19.7 TABLE S2 Pangenome-wide association studies with Scoary. Download Table S2, XLSX file, 0.04 MB. Copyright © 2019 Yuan et al. 2019 Yuan et al. This content is distributed under the terms of the Creative Commons Attribution 4.0 International license.

## DISCUSSION

Through sequencing and analysis of the genomes of eight intraocular isolates of *Bacillus* spp., we have gained insight into the phylogenetic evolution and molecular signatures of intraocular pathogens. To our knowledge, this is the first and largest collection of genomic sequences of bacterial isolates from patients with *Bacillus* endophthalmitis. Although some case studies covering whole-genome isolates of bacterial pathogens in endophthalmitis have been performed (26, 27), this study uniquely focused on opportunistic *Bacillus* pathogens that cause devastating eye infections. Initial bacterial isolates were sampled in 2010, and sampling was continued, allowing us to obtain a comprehensive picture of the genetic alterations and adaptability of microorganisms to infect the eye and to facilitate future studies of endophthalmitis epidemiology and therapeutics.

In this study, we defined the genomic features and phylogenetic diversity of *Bacillus* spp. causing devastating endophthalmitis. All genomes revealed high quality and similar benchmark data, including genome size, GC content, and the number of predicted genes (see Table S1 in the supplemental material). The completeness of each whole-genome sequencing was more than 99%, and we have low contamination levels in our sequencing libraries. The high coverage of our genome assembly helped obtain complete and accurate genome and gene analysis. Phylogenetic analysis and intravitreal injections showed that the intraocular isolates belong to B. cereus, B. thuringiensis, and a novel strain of *B. toyonensis* which was closely related to Bacillus toyonensis sp. nov., a new species of the Bacillus cereus group (28). Interestingly, the phylogenetic position of Bacillus cereus LY7 is closely related to the gastrointestinal isolate RIMV/BC/126 (29), which was originally submitted as Bacillus cereus but which was modified in August 2018 due to the average nucleotide identity (ANI) data of Bacillus wiedmannii strain FCC41 (30). This further indicates the increasing application of whole-genome sequences to discriminate information and provide taxonomic identification that surpasses previous typing methods. These observations highlight the need for further studies regarding the genomics and epidemiology of *Bacillus* spp. in endophthalmitis.

It is well established that *Bacillus* spp. produce a range of tissue-destructive exotoxins that contribute to devastating outcomes in endophthalmitis (4–8). However, recent studies into the pathogenesis of *Bacillus*-induced endophthalmitis have identified several other factors that contribute to the poor outcome of *Bacillus* endophthalmitis. Through whole-genome analysis, we have provided a robust framework to redefine the species clusters of this genus, mapping genetic traits and scoring the significance of pathogenic genes based on their presence or absence. Through analysis of the genes gained or lost from intraocular lineages, it is clear that four virulence factors (*plcA-2*, *InhA-3*, *InhA-4*, and *hblA-5*) and a flagellar cap protein, FliD (*fliD*), are overrepresented in intraocular isolates compared to gastrointestinal isolates. This comparison should be interpreted with caution since it was underpowered (8 intraocular isolates versus 15 gastrointestinal isolates). However, the function of these genes may be related to the ability of intraocular strains to cause devastating endophthalmitis. In addition, to investigate the expression and function of *plcA-2*, we used pET-28a expression vectors to express recombinant His-tagged *plcA-2* in Escherichia coli and performed purifications using the Äkta pure chromatography system (see Fig. S5 in the supplemental material). Phosphatidylinositol-specific phospholipase Cs (PI-PLCs) are secreted by various Gram-positive bacterial pathogens, including *Bacillus* spp. and Listeria monocytogenes (31). Due to the different protein structures and alternative cleavage activity of glycosyl-PI (GPI)-anchored proteins (24, 32), PI-PLCs from different species dysregulate the immune response through different mechanisms: *Bacillus* enzymes downmodulate dendritic cell function and T-cell responses (33), while enzymes from L. monocytogenes reduce phosphatidylinositol (3,4,5)-trisphosphate (PIP3) levels on preautophagosomal structures, leading to the prevention of autophagic flux, favoring the escape of cytosolic bacteria from host autophagic defenses (34). Metallopeptidase InhA, the major component of the exosporium, is essential for efficient spore escape from macrophages through permeability alterations in the macrophage membranes that liberate the mature form of the crucial escape effector NprA (35, 36). Although Callegan et al. showed that the role of the BL toxin in intraocular B. cereus infection was limited (8), Beecher et al. demonstrated that purified hemolysin BL (HBL) is highly damaging to retinal tissue *in vitro* and that the intravitreal injection of HBL into rabbits produces symptoms of B. cereus endophthalmitis (4, 5). B. cereus possesses flagella which allow the organism to migrate within the eye during intraocular infection. When B. cereus infects the eye, it rapidly migrates from the initial site of injection into the vitreous and to the anterior segment within 6 to 12 hpi. The absence of motility affects toxin production; hence, nonmotile *Bacillus* is less pathogenic (16, 37). Taken together, the acquisition of *plcA-2*, *InhA-3*, *InhA-4*, *hblA-5*, and *fliD* suggests that escaping host immune responses, particularly from macrophages, increases virulence and may be central to the ability of intraocular strains to cause devastating endophthalmitis. It is often observed that as pathogens acquire virulence determinants, they increasingly adapt to specific hosts (38–40). The application of whole-genome sequencing to the study of host adaptation has revealed remarkable patterns of evolution in *Yersinia* (41), *Klebsiella* (42), *Salmonella* (38, 43), and *Pseudomonas* (44). *Bacillus* is of particular importance as it gains functional genes during host infection within intraocular-pathogenic lineages. Thus, the dramatic genetic changes demonstrated across the different lineages form a paradigm rather than accidental events and appear to emphasize the emergence of pathogenic “ocular bacteria.”

10.1128/mSystems.00745-19.5 FIG S5 Purification of *plcA-2* and His-tagged recombinant protein. Lane 1 (before induction), expression of recombinant protein without induction; lane 2 (after induction), expression of recombinant protein after induction; lane 3 (flowthrough), the supernatant passes through the eluent behind the HisTrap HP 1-ml column; lanes 4 and 5 (supernatant and precipitation), after induction, the liquid was broken up under high pressure and centrifuged into supernatant and precipitate; lane 6 (purified protein 1), recombinant protein (the solution is the elution buffer); lane 7 (purified protein 2), the recombinant protein goes through a column of desalination (the solution is the equilibrium liquid); lane 8 (marker), dual-color prestained protein marker from Epizyme. Download FIG S5, JPG file, 0.5 MB. Copyright © 2019 Yuan et al. 2019 Yuan et al. This content is distributed under the terms of the Creative Commons Attribution 4.0 International license.

### Conclusions.

In this study, we reveal how a clinical collection of bacteria sampled from acutely infected patients constitutes a valuable basis for understanding the pathogenicity and evolution of pathogens *in vivo*. To our knowledge, this is the first report of *Bacillus* isolates from human intraocular infections to be sequenced, and *B. toyonensis* strain LY557 has the ability to cause complete retinal function loss, in a manner comparable to B. thuringiensis and B. cereus. Notably, we identified several molecular signatures of virulence and motility genes that were overrepresented in intraocular isolates, which may facilitate host adaption. Sampling should now be continued, as the results obtained facilitate comparative studies as more sequencing data sets become available. This may aid the design of future intervention strategies in the clinical setting.

## MATERIALS AND METHODS

### Clinical characteristics.

Three patients’ eyeballs were observed, and all underwent complete ophthalmic examinations and B-scan ultrasonography. Vitreous smears were stained with Gram’s solution. Colony morphological and bacterial microstructures were observed after culturing on blood plates at 37°C for 24 h.

### Bacterial strains and experimental bacterial endophthalmitis.

The representative strains used in experimental mouse endophthalmitis models included *Bacillus* sp. strains LY557, LY178, and LY7, which are clinical strains isolated from patients diagnosed with posttraumatic endophthalmitis. Three inoculated strains were cultured for 6 h at 37°C and 250 rpm and harvested during the log phase of growth. All female wild-type C57BL/6 mice, which have no retinal degeneration such as rd1 and rd8, were purchased from the Animal Center of Wenzhou Medical University (Wenzhou, China). Mice were kept in a constant-temperature environment with a 12-h on-or-off cycle and provided water and a normal diet. For the experiments, 6- to 8-week-old female C57BL/6 mice were anesthetized with a combined preparation of ketamine (70 mg/kg body weight) and xylazine (5 mg/kg body weight). Experimental bacterial endophthalmitis models were established by injecting 1 μl of phosphate-buffered saline (PBS; pH 7.4) containing 100 CFU bacteria into the vitreous chamber of the eyes. Contralateral eyeballs were injected with 1 μl of sterile PBS as a control group.

### Histopathological analysis.

Infected eyeballs and contralateral eyeballs were harvested from each mouse at 3, 6, 9, and 12 hpi. Each eyeball was incubated in Davison’s fixative for 36 h at room temperature. Eyeballs were dehydrated using graded ethanol, paraffin embedded, sectioned, and stained with H&E. Sections are representative of at least 3 eyes observed at each time point from a minimum of 3 independent experiments.

### Electroretinography.

ERG was used to detect retinal function in the mice infected with bacteria at different time points. After dark adaptation, mice were anesthetized with ketamine (70 mg/kg body weight) and xylazine (5 mg/kg body weight) under a dim red light. A gold-wire loop electrode was placed over the cornea once the pupils were dilated. Full-field ERGs were performed using a standard Ganzfeld dome stimulating and recording system (Roland Consult, Wiesbaden, Germany). The potential response of each layer of the retina changed after a transient flash of white light (1,200 cd s m^−2^). A-wave and B-wave amplitudes were recorded, and retinal function was assessed as follows: 100% − {[1 − (experimental A-wave or B-wave amplitude/control A-wave or B-wave amplitude)] × 100%}. Values represent means ± SEM for *n* ≥ 6 eyes at each time point from ≥3 independent experiments.

### Inflammatory mediator expression.

The expression levels of inflammatory mediators in infected and contralateral eyeballs were detected by enzyme-linked immunosorbent assays (ELISAs). Each eyeball was harvested at the indicated time points and homogenized in sterile PBS containing protease inhibitor cocktail (Bimake, Houston, TX, USA). Concentrations of IL-6, TNF-α, and IL-1β were estimated using mouse ELISA kits (Dakewe, Shenzhen, Guangdong, China). The lower limits of detection for the ELISAs were as follows: TNF-α, 15.6 pg/ml; IL-1β, 31.3 pg/ml; and IL-6, 15.6 pg/ml. Values represent means ± SEM for *n* ≥ 6 eyes at each time point from ≥3 independent experiments.

### Genome sequencing and assembly.

Genomic DNA of the eight intraocular isolates was prepared from purified bacterial cultures using the TruSeq Nano DNA HT Sample prep kit and sequenced on an Illumina HiSeq X platform, generating 150-bp paired-end reads. Sequence reads from each isolate were *de novo* assembled using SPAdes v.3.13 (45). To evaluate the quality of each assembly, we used QUAST v5.0.2, BBMap, and CheckM v1.0.18. Publicly available sequences for 125 *Bacillus* reported isolates were downloaded from GenBank. To obtain uniform and consistent annotations for core and pangenome analyses, all sequences were annotated using Prokka v.1.12 (46).

### Core-genome identification and phylogenetic analysis.

Two core genomes were constructed. The first contained proteins common to all B. cereus genomes, while the second contained proteins common to intraocular isolate-related genomes. Pangenome estimates for the study genomes were performed using Roary v.3.12 (-e -n -i 90 -cd 99) (47), with a minimum percent identity for blastp of 90% and an alignment of all core genes (present in 99% of isolates). Single nucleotide polymorphisms (SNPs) in the core genes were extracted and used to construct a maximum likelihood tree using RAxML with 100 bootstraps and a midpoint root (-s -f a -x 12345 -p 12345 -N 100 -n core -T 4 -m GTRCAT). We used RAMI (20) to analyze the ML tree of 129 genomes and identified distinct clusters based on patristic distance (*P = *0.01, i.e., branch length between isolates).

### Comparative analysis of virulence genes.

Putative virulence genes were collated from the Virulence Factors of the Pathogenic Bacteria Database (21). Intraocular pathogen-associated studies of all annotated virulence genes were performed using Fisher’s exact tests and Bonferroni’s correction of *P* values. An alignment for each of the extracted candidate virulence determinant genes was constructed using Clustal Omega (48) and visualized using ESPript (49).

### Statistical analysis.

Statistical analyses were performed in R. PCA was performed using the prcomp function. Differences in the total number of unique genes between distinct lineages and acquired virulence genes between intraocular and gastrointestinal isolates were assessed using Wilcoxon rank sum tests. We performed pangenome-wide association studies (PGWAS) using Scoary v1.6.16 (-g -t -c I -p 0.05).

### Ethics approval and consent to participate.

The study was approved by the ethics committee of the Eye Hospital of Wenzhou Medical University (approval number KYK[2015]34). The use of 6- to 8-week-old female C57BL/6 mice was approved by the Wenzhou Medical University Animal Policy and Welfare Committee (no. wydw2018-0006) according to “The Detailed Rules and Regulations of Medical Animal Experiments Administration and Implementation” (document no. 1998-55, Ministry of Public Health, People’s Republic of China).

### Data availability.

The sequence data obtained in this study have been submitted to the NCBI Sequence Read Archive (SRA) under SRA accession number PRJNA566061. The data, code, and analyses are available at https://github.com/DanielWYuan/Bacillus_Endophthalmitis.
