# Supplementary figures and images for: Molecular Signatures Related to the Virulence of Bacillus cereus Sensu Lato, a Leading Cause of Devastating Endophthalmitis
Source: mSystems. 2019 Dec 3;4(6):e00745-19. doi: 10.1128/mSystems.00745-19 (PMC6890933; doi:10.1128/mSystems.00745-19)

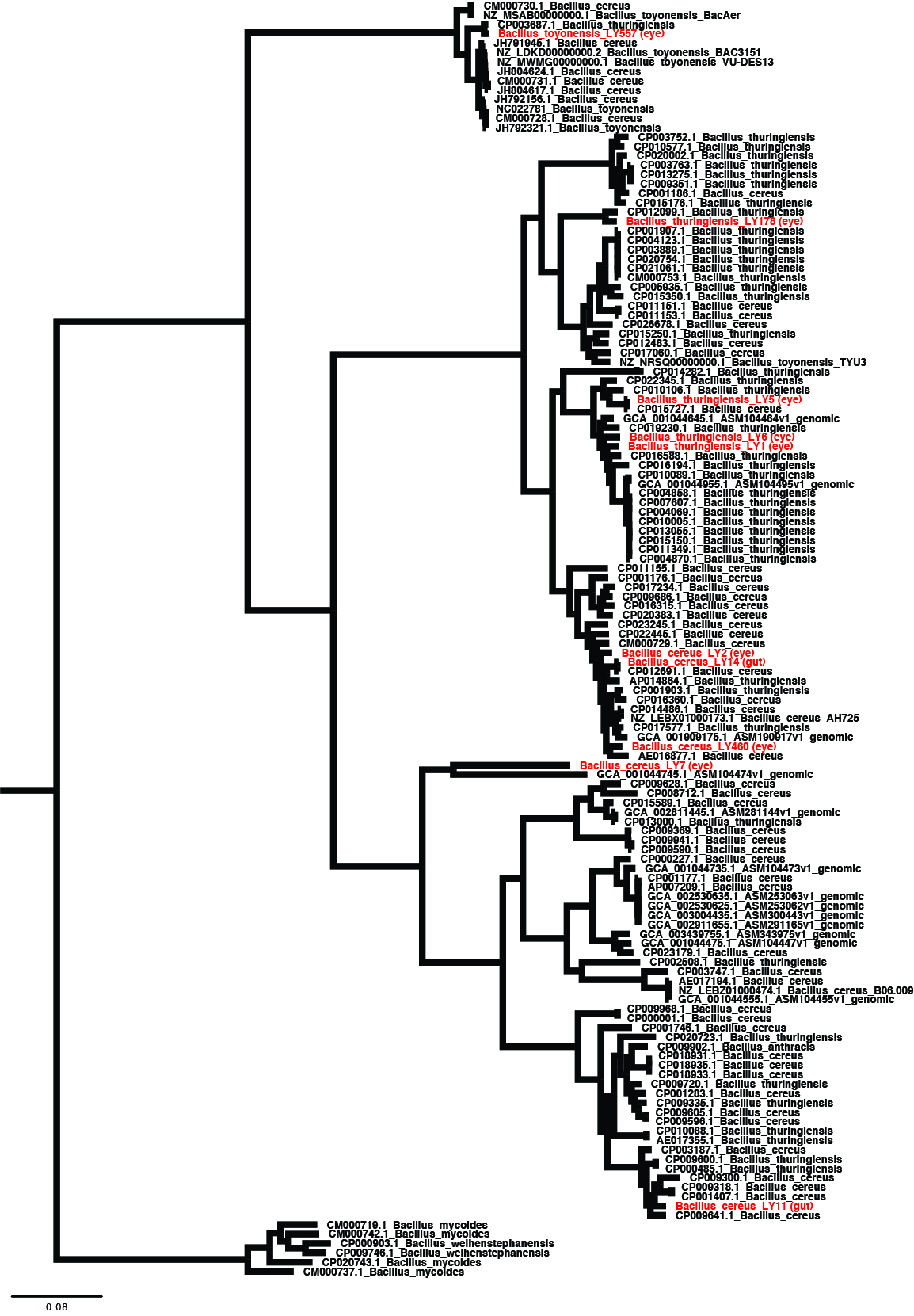

Supplement: FIG S1 [file mSystems.00745-19-sf001.jpg]

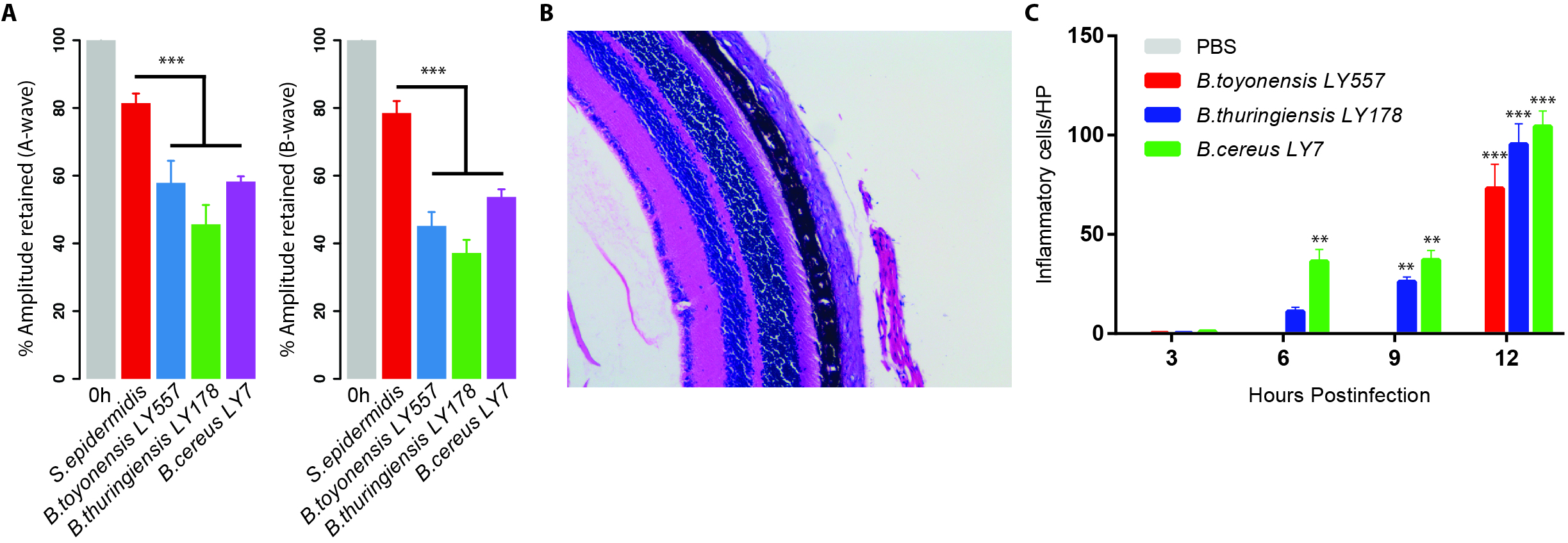

Supplement: FIG S2 [file mSystems.00745-19-sf002.jpg]

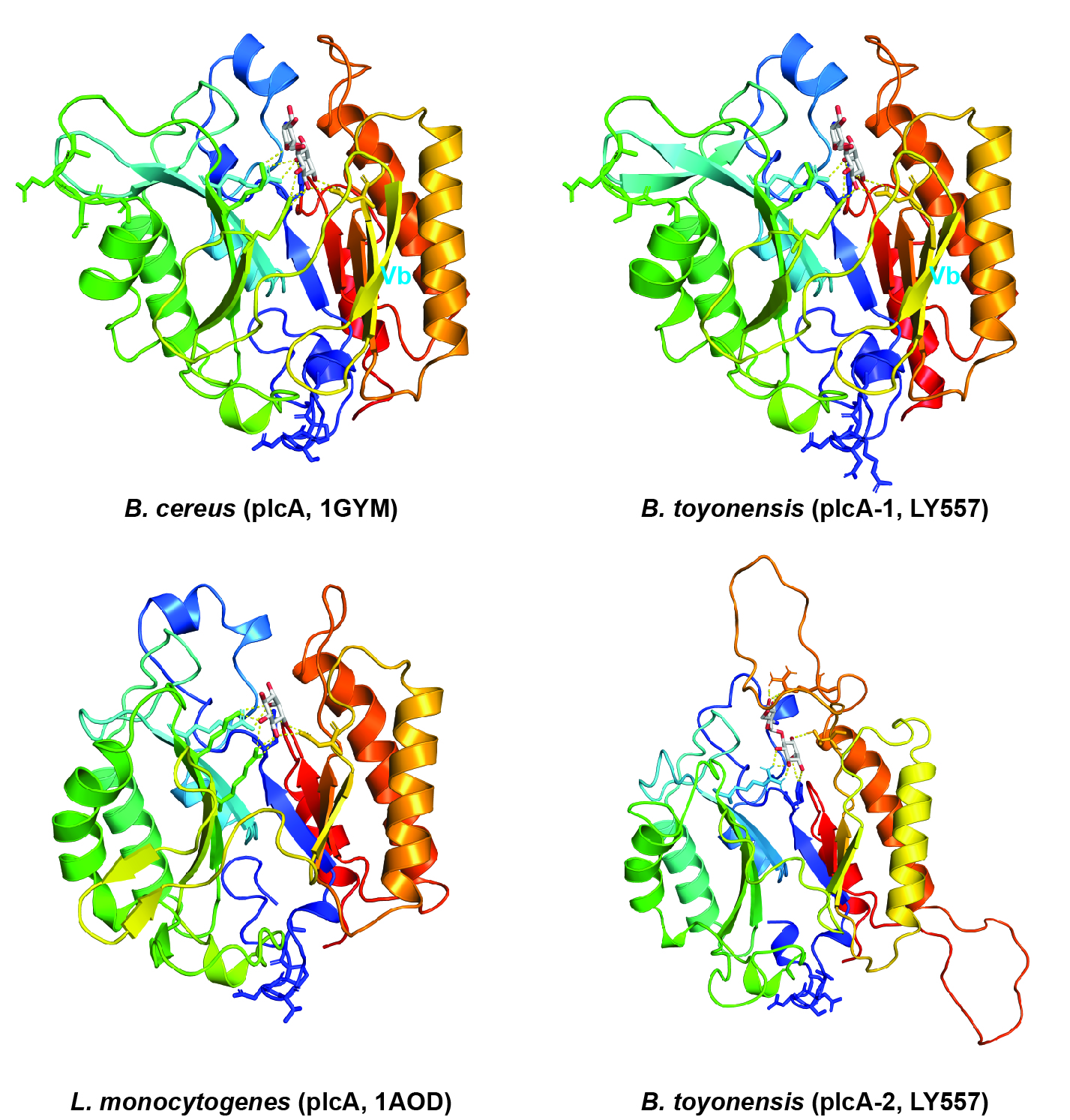

Supplement: FIG S4 [file mSystems.00745-19-sf004.jpg]

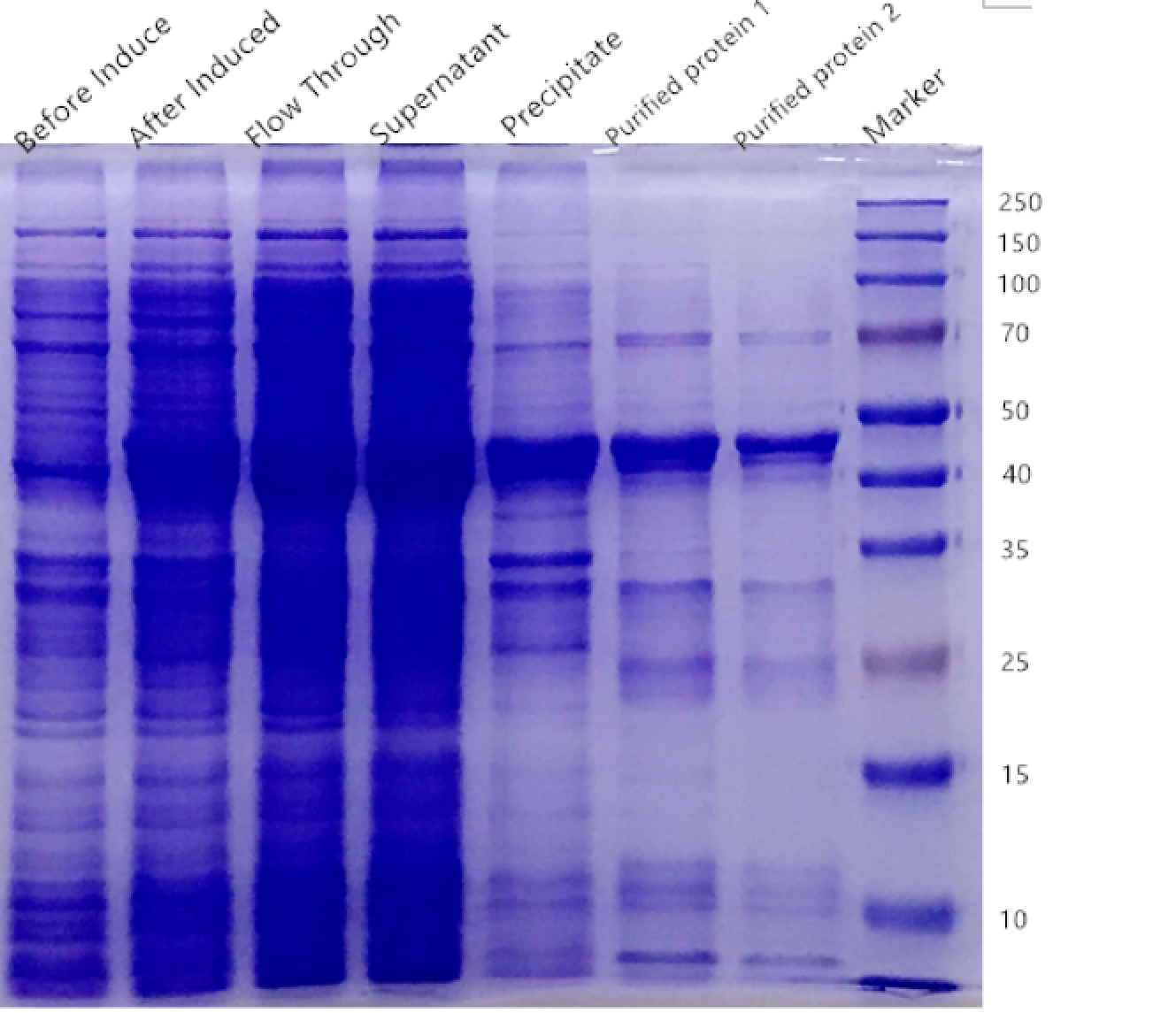

Supplement: FIG S5 [file mSystems.00745-19-sf005.jpg]
