# Supplementary material for: Molecular Signatures Related to the Virulence of Bacillus cereus Sensu Lato, a Leading Cause of Devastating Endophthalmitis
Source: mSystems. 2019 Dec 3;4(6):e00745-19. doi: 10.1128/mSystems.00745-19 (PMC6890933; doi:10.1128/mSystems.00745-19)
Supplement: FIG S3 [file mSystems.00745-19-sf003.pdf]

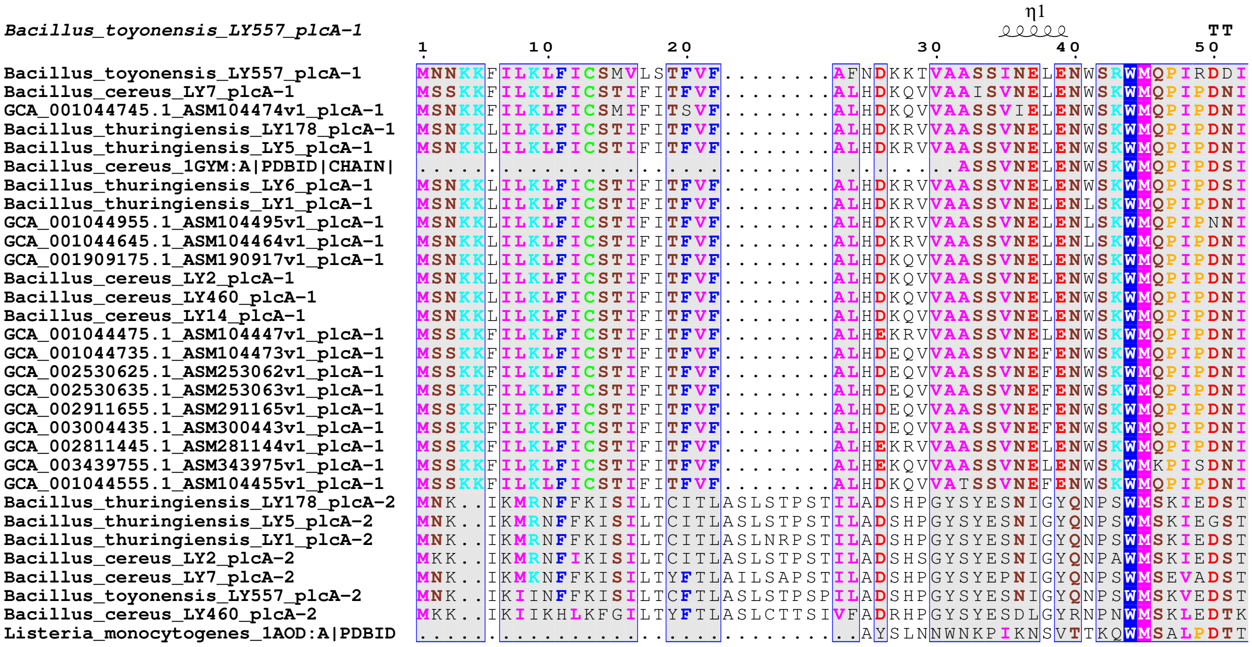
Bacillus_toyonensis_LY557_plcA-1
η1
TT
1 10 20 30 40 50
Bacillus_toyonensis_LY557_plcA-1
Bacillus_cereus_LY7_plcA-1
GCA_001044745.1_ASM104474v1_plcA-1
Bacillus_thuringiensis_LY178_plcA-1
Bacillus_thuringiensis_LY5_plcA-1
Bacillus_cereus_1GYM:A|PDBID|CHAIN|
Bacillus_thuringiensis_LY6_plcA-1
Bacillus_thuringiensis_LY1_plcA-1
GCA_001044955.1_ASM104495v1_plcA-1
GCA_001044645.1_ASM104464v1_plcA-1
GCA_001909175.1_ASM190917v1_plcA-1
Bacillus_cereus_LY2_plcA-1
Bacillus_cereus_LY460_plcA-1
Bacillus_cereus_LY14_plcA-1
GCA_001044475.1_ASM104447v1_plcA-1
GCA_001044735.1_ASM104473v1_plcA-1
GCA_002530625.1_ASM253062v1_plcA-1
GCA_002530635.1_ASM253063v1_plcA-1
GCA_002911655.1_ASM291165v1_plcA-1
GCA_003004435.1_ASM300443v1_plcA-1
GCA_002811445.1_ASM281144v1_plcA-1
GCA_003439755.1_ASM343975v1_plcA-1
GCA_001044555.1_ASM104455v1_plcA-1
Bacillus_thuringiensis_LY178_plcA-2
Bacillus_thuringiensis_LY5_plcA-2
Bacillus_thuringiensis_LY1_plcA-2
Bacillus_cereus_LY2_plcA-2
Bacillus_cereus_LY7_plcA-2
Bacillus_toyonensis_LY557_plcA-2
Bacillus_cereus_LY460_plcA-2
Listeria_monocytogenes_1AOD:A|PDBID

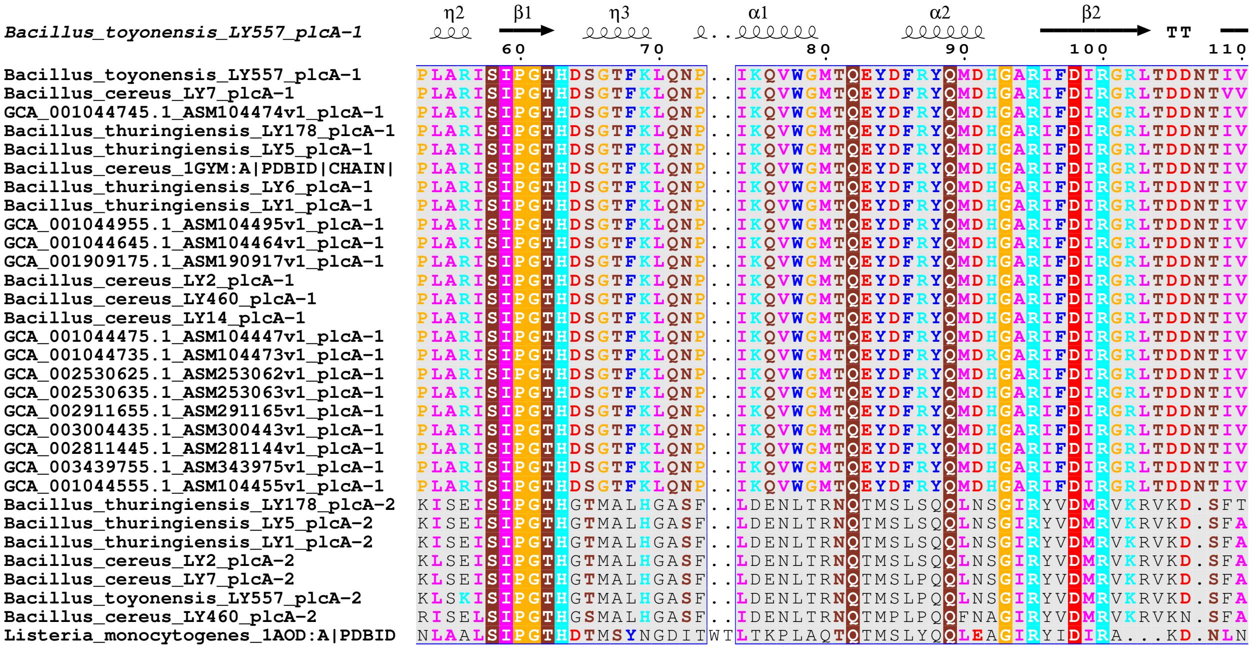
Bacillus_toyonensis_LY557_plcA-1
η2 β1 η3 α1 α2 β2
TT
60 70 80 90 100 110
Bacillus_toyonensis_LY557_plcA-1
Bacillus_cereus_LY7_plcA-1
GCA_001044745.1_ASM104474v1_plcA-1
Bacillus_thuringiensis_LY178_plcA-1
Bacillus_thuringiensis_LY5_plcA-1
Bacillus_cereus_1GYM:A|PDBID|CHAIN|
Bacillus_thuringiensis_LY6_plcA-1
Bacillus_thuringiensis_LY1_plcA-1
GCA_001044955.1_ASM104495v1_plcA-1
GCA_001044645.1_ASM104464v1_plcA-1
GCA_001909175.1_ASM190917v1_plcA-1
Bacillus_cereus_LY2_plcA-1
Bacillus_cereus_LY460_plcA-1
Bacillus_cereus_LY14_plcA-1
GCA_001044475.1_ASM104447v1_plcA-1
GCA_001044735.1_ASM104473v1_plcA-1
GCA_002530625.1_ASM253062v1_plcA-1
GCA_002530635.1_ASM253063v1_plcA-1
GCA_002911655.1_ASM291165v1_plcA-1
GCA_003004435.1_ASM300443v1_plcA-1
GCA_002811445.1_ASM281144v1_plcA-1
GCA_003439755.1_ASM343975v1_plcA-1
GCA_001044555.1_ASM104455v1_plcA-1
Bacillus_thuringiensis_LY178_plcA-2
Bacillus_thuringiensis_LY5_plcA-2
Bacillus_thuringiensis_LY1_plcA-2
Bacillus_cereus_LY2_plcA-2
Bacillus_cereus_LY7_plcA-2
Bacillus_toyonensis_LY557_plcA-2
Bacillus_cereus_LY460_plcA-2
Listeria_monocytogenes_1AOD:A|PDBID

```
                                      β3    β4          α3                    β5                α4
Bacillus_toyonensis_LY557_plcA-1      →TT───→ ℓℓℓℓℓℓℓℓℓℓℓℓℓℓ      ─────→         TT      ℓℓℓℓℓ......ℓ
                                           120       130       140       150       160

Bacillus_toyonensis_LY557_plcA-1      LHHGPLYLYVTLHEFINEAKQFLKDNPSETIIMSLKKEYEDMKGAETSFSSTF......E
Bacillus_cereus_LY7_plcA-1            LHHGPLYLYVTMHEFINEAKQFLKDNPSETIIMSLKKEYEDMKGAEGSFSSTF......E
GCA_001044745.1_ASM104474v1_plcA-1    LHHGPLFLYVTLHEFINEAKQFLKDNPSETIIMSLKKEYEDMKGAEGSFSNTF......E
Bacillus_thuringiensis_LY178_plcA-1   LHHGPLYLYVTLHEFINEAKQFLKDNPSETIIMSLKKEYEDMKGAEGSFISTF......E
Bacillus_thuringiensis_LY5_plcA-1     LHHGPLYLYVTLHEFINEAKQFLKDNPSETIIMSLKKEYEDMKGAEGSFSSTF......E
Bacillus_cereus_1GYM:A|PDBID|CHAIN|   LHHGPLYLYVTLHEFINEAKQFLKDNPSETIIMSLKKEYEDMKGAEDSFSSTF......E
Bacillus_thuringiensis_LY6_plcA-1     LHHGPLYLYVTLHEFINEAKQFLKDNPSETIIMSLKKEYEDMKGAEDSFSSTF......L
Bacillus_thuringiensis_LY1_plcA-1     LHHGPLYLYVTLHEFINEAKQFLKDNPSETIIMSLKKEYEDMKGAEDSFSSTF......E
GCA_001044955.1_ASM104495v1_plcA-1    LHHGPLYLYVTLHEFINEAKQFLKDNPSETIIMSLKKEYEDMKGAEDSFSSTF......E
GCA_001044645.1_ASM104464v1_plcA-1    LHHGPLYLYVTLHEFINEAKQFLKDNPSETIIMSLKKEYEDMKGAEDSFSSTF......E
GCA_001909175.1_ASM190917v1_plcA-1    LHHGPLYLYVTLHEFINEAKQFLRDNPSETIIMSLKKEYEDMKGAEDSFSSTF......E
Bacillus_cereus_LY2_plcA-1            LHHGPLYLYVTLHEFINEAKQFLRDNPSETIIMSLKKEYEDMKGAEDSFSSTF......E
Bacillus_cereus_LY460_plcA-1          LHHGPLYLYVTLHEFINEAKQFLRDNPSETIIMSLKKEYEDMKGAEDSFSSTF......E
Bacillus_cereus_LY14_plcA-1           LHHGPLYLYVTLHEFINEAKQFLRDNPSETIIMSLKKEYEDMKGAEDSFSSTF......E
GCA_001044475.1_ASM104447v1_plcA-1    LHHGPLYLYVTLHEFINEAKKFLKDNPSETIIMSLKKEYEDMKGAEGSFSNTF......E
GCA_001044735.1_ASM104473v1_plcA-1    LHHGPLYLYVTLHEFINEAKQFLKDNPSETIIMSLKKEYEDMKGAEGSFSNTF......E
GCA_002530625.1_ASM253062v1_plcA-1    LHHGPLYLYVTLHEFINEAKQFLKDNPSETIIMSLKKEYEDMKGAEGSFSNTF......E
GCA_002530635.1_ASM253063v1_plcA-1    LHHGPLYLYVTLHEFINEAKQFLKDNPSETIIMSLKKEYEDMKGAEGSFSNTF......E
GCA_002911655.1_ASM291165v1_plcA-1    LHHGPLYLYVTLHEFINEAKQFLKDNPSETIIMSLKKEYEDMKGAEGSFSNTF......E
GCA_003004435.1_ASM300443v1_plcA-1    LHHGPLYLYVTLHEFINEAKQFLKDNPSETIIMSLKKEYEDMKGAEGSFSNTF......E
GCA_002811445.1_ASM281144v1_plcA-1    LHHGPLYLYVTLHEFINEAKQFLKDNPSETIIMSLKKEYEDMKGAEGSFSNVF......E
GCA_003439755.1_ASM343975v1_plcA-1    LHHGPLYLYVTLHEFINEAKQFLKDNPSETIIMSLKKEYEDMKGAEGSFSNTF......E
GCA_001044555.1_ASM104455v1_plcA-1    LHHGPLYLYVTLHEFINEAKQFLKDNPSETIIMSLKKEYEDMKGAESSFSNTF......E
Bacillus_thuringiensis_LY178_plcA-2   MYHGVVNQKAVFEDVLKETIQFLKDHPTETILMRLKEETTPESGSL.SFEEIFLKYKNVN
Bacillus_thuringiensis_LY5_plcA-2     MYHGVVNQKAVFEDVLKETIQFLKDHPTETILMRLKEETNPESGSL.SFEEIFLKYKNVN
Bacillus_thuringiensis_LY1_plcA-2     MYHGVVNQKAVFEDVLKETIQFLKDHPTETILMRLKEETTPESGSL.SFEEIFLKYKNVN
Bacillus_cereus_LY2_plcA-2            MYHGIVNQKAVFEDVLKETIQFLKDYPTETILMRLKEETTPESGSL.SFEEIFLKYKNVN
Bacillus_cereus_LY7_plcA-2            MYHGIVNQKAVFEDVLKDAIQFLKDYPTETILMRLKEETTPESGSP.SFEEIFLKYKNVN
Bacillus_toyonensis_LY557_plcA-2      MYHGIVNQKAMFEDVLKEAIQFLKDYPTETILMRLKEETTPESGSL.SFEEIFLKYKNVN
Bacillus_cereus_LY460_plcA-2          MQHGIVNQKAMFEDVLKETIQFLRENPQETILMRLKEETDPENGSQ.SFEEIFKSYKDRN
Listeria_monocytogenes_1AOD:A|PDBID   IYHGPIFLNASLSGVLETITQFLKKNPKETIIMRLKDEQNSND....SFDYRIQPLINIY


                                                        α5      β6                 β7
Bacillus_toyonensis_LY557_plcA-1      ℓℓ     TT             ℓℓℓℓ   ────→            TT───→
                                          170       180       190       200       210       220

Bacillus_toyonensis_LY557_plcA-1      KNYFRDPIFL..KTEGNIKLGDARGKIVLLKRYSGSNDSGGYNNFYWPDNETFTSTINQN
Bacillus_cereus_LY7_plcA-1            KKYFVDPIFL..KTDGNIKLGDARGKIVLLKRYSGSNESGGYNNFYWPDNETFTTTVNQN
GCA_001044745.1_ASM104474v1_plcA-1    KNYFVDPIFL..KTEGNIKLGDARGKIVLLKRYSGSNESGGYNNFYWPDNETFTTTVNQN
Bacillus_thuringiensis_LY178_plcA-1   KKYFVDPIFL..KTEGNIKLGDARGKIVLLKRYSGSNESGGYNNFYWPDNETFTTTVNQN
Bacillus_thuringiensis_LY5_plcA-1     KNYFVDPIFL..KTEGNIKLGDARGKIVLLKRYSGSNESGGYNNFYWPDNETFTTTVNQN
Bacillus_cereus_1GYM:A|PDBID|CHAIN|   KKYFVDPIFL..KTEGNIKLGDARGKIVLLKRYSGSNEPGGYNNFYWPDNETFTTTVNQN
Bacillus_thuringiensis_LY6_plcA-1     KKYFVDPIFL..KTEGNIKLGDARGKIVLLKRYSGSNEPGGYNNFYWPDNETFTTTVNQN
Bacillus_thuringiensis_LY1_plcA-1     KKYFVDPIFL..KTEGNIKLGDARGKIVLLKRYSGSNEPGGYNNFYWPDNETFTTTVNQN
GCA_001044955.1_ASM104495v1_plcA-1    KKYFVDPIFL..KTEGNIKLGDARGKIVLLKRYSGSNEPGGYNNFYWPDNETFTTTVNQN
GCA_001044645.1_ASM104464v1_plcA-1    KKYFVDPIFL..KTEGNIKLGDARGKIVLLKRYSGSNEPGGYNNFYWPDNETFTTTVNQN
GCA_001909175.1_ASM190917v1_plcA-1    KNYFVDPIFL..KTEGNIKLGDARGKIVLLKRYSGSNESGGYNNFYWPDNETFTTTVNQN
Bacillus_cereus_LY2_plcA-1            KNYFVDPIFL..KTEGNIKLGDARGKIVLLKRYSGSNESGGYNNFYWPDNETFTTTVNQN
Bacillus_cereus_LY460_plcA-1          KNYFVDPIFL..KTEGNIKLGDARGKIVLLKRYSGSNESGGYNNFYWPDNETFTTTVNQN
Bacillus_cereus_LY14_plcA-1           KNYFVDPIFL..KTEGNIKLGDARGKIVLLKRYSGSNESGGYNNFYWPDNETFTTTVNQN
GCA_001044475.1_ASM104447v1_plcA-1    KNYFVDPIFL..KTEGNIKLGDARGKIVLLKRYGGSNESGGYNNFYWPDNEMFTTTVNQN
GCA_001044735.1_ASM104473v1_plcA-1    KNYFVDPIFL..KTEGNIKLGDARGKIVLLKRYGGSNESGGYNNFYWPDNETFTTTVNHN
GCA_002530625.1_ASM253062v1_plcA-1    KNYFVDPIFL..KTEGNIKLGDARGKIVLLKRYGGSNESGGYNNFYWPDNETFTTTVNHN
GCA_002530635.1_ASM253063v1_plcA-1    KNYFVDPIFL..KTEGNIKLGDARGKIVLLKRYGGSNESGGYNNFYWPDNETFTTTVNHN
GCA_002911655.1_ASM291165v1_plcA-1    KNYFVDPIFL..KTEGNIKLGDARGKIVLLKRYGGSNESGGYNNFYWPDNETFTTTVNHN
GCA_003004435.1_ASM300443v1_plcA-1    KNYFVDPIFL..KTEGNIKLGDARGKIVLLKRYGGSNESGGYNNFYWPDNETFTTTVNHN
GCA_002811445.1_ASM281144v1_plcA-1    KKYFVDPIFL..KTEGNIKLGDARGKIVLLKRYGGSNESGGYNNFYWPDNETFTTTVNQN
GCA_003439755.1_ASM343975v1_plcA-1    KNYFVDPIFL..KTEGNIKLGDARGKIVLLKRYGGSNESGGYNNFYWSDNEMFTTTVNQN
GCA_001044555.1_ASM104455v1_plcA-1    KSYFVDPIFL..KTEGNIKLGDARGKIVLLKRYGGSNESGGYNNFYWPDNETFTTTVNQN
Bacillus_thuringiensis_LY178_plcA-2   ASYFWDPNSVPTSDRNNPTLGGTRGKIVILQNFTSAQLYGID...YEGLN..........
Bacillus_thuringiensis_LY5_plcA-2     ASYFWDPNSVPTSDRNNPTLGDTRGKIVILQNFTSAQLYGID...YEGLN..........
Bacillus_thuringiensis_LY1_plcA-2     ASYFWDPNSVPTSDRNNPTLGDTRGKIVILQNFTSAQLYGID...YEGLN..........
Bacillus_cereus_LY2_plcA-2            ASYFWDPNSVPTSDRNNPTLGDTRGKIVILQNFTSTQLYGID...YEGLN..........
Bacillus_cereus_LY7_plcA-2            ASYFWDPNSVPTIDKNNPTLGDIRGKIVVLQNFTSSQLYGID...YDSLN..........
Bacillus_toyonensis_LY557_plcA-2      ASYFWDPNSVQTSDRNNPTLGDIRGKIVILQNFTSSQLYGID...YESLN..........
Bacillus_cereus_LY460_plcA-2          VSYFWEPNSVPFSERNNPKLGDIRGKIVILQNFSAVEEYGIN...YESLN..........
Listeria_monocytogenes_1AOD:A|PDBID   KDYFYTTPRTD.TSNKIPTLKDVRGKILLLSENHTKKPLVIN...SRKFGMQF.....GA
```

```
                                        β8               α6                  β9
Bacillus_toyonensis_LY557_plcA-1        230       240       250       260       270
Bacillus_toyonensis_LY557_plcA-1        VNVTVQDKYKVSYD.....EKVNAIKDTINETINNSEDVNHLYINFTSLSSGGTA.WNSP
Bacillus_cereus_LY7_plcA-1              VNVTVQDKYKVSYD.....EKVKSIKDTINETMNNSEDLNHLYINFTSLSSGGTA.WNSP
GCA_001044745.1_ASM104474v1_plcA-1      INVTVQDKYKVSYD.....EKVKSIKETIKETINNSEDLNHLYINFTSLSSGGTA.WNSP
Bacillus_thuringiensis_LY178_plcA-1     VNVTVQDKYKVNYD.....EKVKSIKDTMDETMNNSEDLNHLYINFTSLSSGGTA.WNSP
Bacillus_thuringiensis_LY5_plcA-1       VNVTVQDKYKVNYD.....EKVKSIKDTMDETMNNSEDLNHLYINFTSLSSGGTA.WNSP
Bacillus_cereus_1GYM:A|PDBID|CHAIN|     ANVTVQDKYKVSYD.....EKVKSIKDTMDETMNNSEDLNHLYINFTSLSSGGTA.WNSP
Bacillus_thuringiensis_LY6_plcA-1       ANVTVQDKYKVSYD.....EKVKSIKDTMDETMNNSEDLNHLYINFTSLSSGGTA.WNSP
Bacillus_thuringiensis_LY1_plcA-1       ANVTVQDKYKVSYD.....EKVKSIKDTMDETMNNSEDLNHLYINFTSLSSGGTA.WNSP
GCA_001044955.1_ASM104495v1_plcA-1      ANVTVQDKYKVSYD.....EKVKSIKDTMDETMNNSEDLNHLYINFTSLSSGGTA.WNSP
GCA_001044645.1_ASM104464v1_plcA-1      ANVTVQDKYKVSYD.....EKVKSIKDTMDETMNNSEDLNHLYINFTSLSSGGTA.WNSP
GCA_001909175.1_ASM190917v1_plcA-1      VNVTVQDKYKVSYD.....EKVKSIKDTMNETMNNSEDLNHLYINFTSLSSGGTA.WNSP
Bacillus_cereus_LY2_plcA-1              VNVTVQDKYKVSYD.....EKVKSIKDTMNETMNNSEDLNHLYINFTSLSSGGTA.WNSP
Bacillus_cereus_LY460_plcA-1            VNVTVQDKYKVSYD.....EKVKSIKDTMNETMNNSEDLNHLYINFTSLSSGGTA.WNSP
Bacillus_cereus_LY14_plcA-1             VNVTVQDKYKVSYD.....EKVKSIKDTMNETMNNSEDLNHLYINFTSLSSGGTA.WNSP
GCA_001044475.1_ASM104447v1_plcA-1      VNVTVQDKYKVGYD.....EKVKSIKDTINETMNNSEDLHHLYINFASLSSGGTA.WNSP
GCA_001044735.1_ASM104473v1_plcA-1      VNVTVQDKYKVGYD.....EKVKSIKDTINETMNNSEDLHHLYINFASLSSGGTA.WNSP
GCA_002530625.1_ASM253062v1_plcA-1      VNVTVQDKYKVGYD.....EKVKSIKDTINETMNNSEDLHHLYINFASLSSGGTA.WNSP
GCA_002530635.1_ASM253063v1_plcA-1      VNVTVQDKYKVGYD.....EKVKSIKDTINETMNNSEDLHHLYINFASLSSGGTA.WNSP
GCA_002911655.1_ASM291165v1_plcA-1      VNVTVQDKYKVGYD.....EKVKSIKDTINETMNNSEDLHHLYINFASLSSGGTA.WNSP
GCA_003004435.1_ASM300443v1_plcA-1      VNVTVQDKYKVGYD.....EKVKSIKDTINETMNNSEDLHHLYINFASLSSGGTA.WNSP
GCA_002811445.1_ASM281144v1_plcA-1      VNVTVQDKYKVGYD.....EKVKSIKDTINETMNNSEDLHHLYINFTSLSSGGTA.WNSP
GCA_003439755.1_ASM343975v1_plcA-1      VNVTVQDKYKVGYD.....EKVKSIKDTINETMNNSEDLHHLYINFTSLSSGGTA.WNSP
GCA_001044555.1_ASM104455v1_plcA-1      VHVTVQDKYKVSYD.....EKVKSIKDTINETMNDSEDLNHLYINFTSLSSGGTA.WNSP
Bacillus_thuringiensis_LY178_plcA-2     ....IQDKFEIGSGPDEIYTKWIAIKNHLHN.TNINFNNGKIYLNHFSGTGGAAAFLNNV
Bacillus_thuringiensis_LY5_plcA-2       ....IQDKFEIGSGPDEIYTKWIAIKNHLQN.TNINFNTRKIYLNHFSGTGGAAAFLNNV
Bacillus_thuringiensis_LY1_plcA-2       ....IQDKFEIGSGPDEIYTKWIAIKNHLQN.TNTNFNNEKIYLNHFSGTGGAAAFLNNV
Bacillus_cereus_LY2_plcA-2              ....IQDKFEIGSGPDEIYAKWIAIKTHLQN.ANTNFNNGKIHLNHFSGTGGAAAFLNNV
Bacillus_cereus_LY7_plcA-2              ....IQDKFEIGSGPNEIYEKWNAIKIHLQS.ANTNFNNGKIYLNHFSGTGGAAAFLNNV
Bacillus_toyonensis_LY557_plcA-2        ....IQDKFEIGSGPDEIYGKWNAVKSHLQN.ANTNFNNGKIYLNHFSGTGGATAFLNNV
Bacillus_cereus_LY460_plcA-2            ....IQDNFEVSAGADGMYEKWSTVKNQLLK.ANNDSNNNHIYLNHFSGTGGATALWNNY
Listeria_monocytogenes_1AOD:A|PDBID     PNQVIQDDYNGPS....VKTKFKEIVQTA...YQASKADNKLFLNHISATSLTFT.....

                                              α7            β10
Bacillus_toyonensis_LY557_plcA-1         280       290       300       310
Bacillus_toyonensis_LY557_plcA-1        YYYASYINPEIANYMKQKNPSRVGWIMQDYINEKWSPL......................
Bacillus_cereus_LY7_plcA-1              YYYASYINPEIAAYIKQEDSKRVGWIIQDYVSDKWSPI......................
GCA_001044745.1_ASM104474v1_plcA-1      YYYASYINPEIAAYIKQEDPKRVGWVIQDYVSDKWSPI......................
Bacillus_thuringiensis_LY178_plcA-1     YYYASYINPEIANDIKQKNPTRVGWVIQDYINEKWSPL......................
Bacillus_thuringiensis_LY5_plcA-1       YYYASYINPEIANYIKQKNPLRVGWVIQDYINEKWSPL......................
Bacillus_cereus_1GYM:A|PDBID|CHAIN|     YYYASYINPEIANYIKQKNPARVGWVIQDYINEKWSPL......................
Bacillus_thuringiensis_LY6_plcA-1       YYYASYINPEIANYIKQKNPLRVGWVIQDYINEKWSPL......................
Bacillus_thuringiensis_LY1_plcA-1       YYYASYINPEIANYIKQKNPLRVGWVIQDYINEKWSPL......................
GCA_001044955.1_ASM104495v1_plcA-1      YYYASYINPEIANYIKQKNPLRVGWVIQDYINEKWSPL......................
GCA_001044645.1_ASM104464v1_plcA-1      YYYASYINPEIANYIKQKNPLRVGWVIQDYINEKWSPL......................
GCA_001909175.1_ASM190917v1_plcA-1      YYYASYINPEIANHIKQKNPARVGWVIQDYINEKWSPL......................
Bacillus_cereus_LY2_plcA-1              YYYASYINPEIANHIKQKNPARVGWVIQDYINEKWSPL......................
Bacillus_cereus_LY460_plcA-1            YYYASYINPEIANHIKQKNPARVGWVIQDYINEKWSPL......................
Bacillus_cereus_LY14_plcA-1             YYYASYINPEIANHIKQKNPARVGWVIQDYINEKWSPL......................
GCA_001044475.1_ASM104447v1_plcA-1      YYYASYINPEIANYIKQKSPTRVGWIIQDYINEKWSPL......................
GCA_001044735.1_ASM104473v1_plcA-1      YYYASYINPEIANYIKQKSPTRVGWIIQDYINEKWSPL......................
GCA_002530625.1_ASM253062v1_plcA-1      YYYASYINPEIANYIKQKSPTRVGWIIQDYINEKWSPL......................
GCA_002530635.1_ASM253063v1_plcA-1      YYYASYINPEIANYIKQKSPTRVGWIIQDYINEKWSPL......................
GCA_002911655.1_ASM291165v1_plcA-1      YYYASYINPEIANYIKQKSPTRVGWIIQDYINEKWSPL......................
GCA_003004435.1_ASM300443v1_plcA-1      YYYASYINPEIANYIKQKSPTRVGWIIQDYINEKWSPL......................
GCA_002811445.1_ASM281144v1_plcA-1      YYYASYINPEIANHIKQKNPTRVGWIIQDYINEKWSPL......................
GCA_003439755.1_ASM343975v1_plcA-1      YYYASYINPEIANYIKQKNPTRVGWIIQDYINEKWSPL......................
GCA_001044555.1_ASM104455v1_plcA-1      YYYASYINPEIANYIKQKNPTRVGWIIQDYINEKWSPL......................
Bacillus_thuringiensis_LY178_plcA-2     YPW..FVASGKESR....NTDSSPKLIQTNSTNAWSDFPRDRNGQVYYGGMNTLGTEL..
Bacillus_thuringiensis_LY5_plcA-2       YPW..FVASGKESR....NTDSSPKLIQTNSTNAWSDFPRDRNGQVYYGGMNTLGTEL..
Bacillus_thuringiensis_LY1_plcA-2       YPW..FVASGKESR....NTDSSPKLIQTNSTNAWSDFPRDRNGQVYYGGMNTLGTEL..
Bacillus_cereus_LY2_plcA-2              YPW..FVASGKESR....NTDSSPKLIQTNSTNAWSDFPRDRNGQVYYGGMNTLGTEL..
Bacillus_cereus_LY7_plcA-2              YPW..FVASGKENR....NTDSNPKLIQTNSTNAWGDFPRDRNGQVYYGGMNTLGTEL..
Bacillus_toyonensis_LY557_plcA-2        YPW..FVASGKENR....NTDSSPKLIQTNFTHEWSDFPRDRSGQIYYGGMNTLGTEL..
Bacillus_cereus_LY460_plcA-2            YPW..FVASGKENR....DTGSNPQLIQKNTTNEWKDFPRDTNGQVFYGGMNTMGTDF..
Listeria_monocytogenes_1AOD:A|PDBID     ......................................PR.....QYAAALNNKVEQFVL
```

```
                                       α8      η4
Bacillus_toyonensis_LY557_plcA-1
                                          320
                                            .
Bacillus_toyonensis_LY557_plcA-1     .LYQEVIRANKSLVKE............................................
Bacillus_cereus_LY7_plcA-1           .LYQEVIRTNKSL...............................................
GCA_001044745.1_ASM104474v1_plcA-1   .LYQEVIRTNKSL...............................................
Bacillus_thuringiensis_LY178_plcA-1  .LYQEVIRANKSLIKE............................................
Bacillus_thuringiensis_LY5_plcA-1    .LYQEVIRANKSLIKE............................................
Bacillus_cereus_1GYM:A|PDBID|CHAIN|  .LYQEVIRANKSLIKE............................................
Bacillus_thuringiensis_LY6_plcA-1    .LYQEVIRANKSLIKE............................................
Bacillus_thuringiensis_LY1_plcA-1    .LYQEVIRANKSLIKE............................................
GCA_001044955.1_ASM104495v1_plcA-1   .LYQEVIRANKSLIKE............................................
GCA_001044645.1_ASM104464v1_plcA-1   .LYQEVIRANKSLIKE............................................
GCA_001909175.1_ASM190917v1_plcA-1   .LYQEVIRANKSLIKE............................................
Bacillus_cereus_LY2_plcA-1           .LYQEVIRANKSLIKE............................................
Bacillus_cereus_LY460_plcA-1         .LYQEVIRANKSLIKE............................................
Bacillus_cereus_LY14_plcA-1          .LYQEVIRANKSLIKE............................................
GCA_001044475.1_ASM104447v1_plcA-1   .LYQEVIRANKSLIRD............................................
GCA_001044735.1_ASM104473v1_plcA-1   .LYQEVIRANKSLIRD............................................
GCA_002530625.1_ASM253062v1_plcA-1   .LYQEVIRANKSLIRD............................................
GCA_002530635.1_ASM253063v1_plcA-1   .LYQEVIRANKSLIRD............................................
GCA_002911655.1_ASM291165v1_plcA-1   .LYQEVIRANKSLIRD............................................
GCA_003004435.1_ASM300443v1_plcA-1   .LYQEVIRANKSLIRD............................................
GCA_002811445.1_ASM281144v1_plcA-1   .LYQEVIRANESLIKE............................................
GCA_003439755.1_ASM343975v1_plcA-1   .LYQEVIRANESLIKE............................................
GCA_001044555.1_ASM104455v1_plcA-1   .LYQEVIRANKSLIKE............................................
Bacillus_thuringiensis_LY178_plcA-2  .LQQGAIKHSGIIAADFPGPGLIDSIIKLNGIHSNEKEILISQISSESSPLSGQQNRSSQ
Bacillus_thuringiensis_LY5_plcA-2    .LQQGAIKHSGIIAADFPGPGLIDSIIKLNGIHSNEKEILISQISSESSPLSGQQNRSSQ
Bacillus_thuringiensis_LY1_plcA-2    .LQQGAIKHSGIIAADFPGPGLIDSIIKLNGIHSNEKEILISQISSESSPLSGQQNRSSQ
Bacillus_cereus_LY2_plcA-2           .LQQGIIKHSGIIAADFPGPGLIDSIIELNGIHSNEKEILISQIPSESSPLSGQHNRSSQ
Bacillus_cereus_LY7_plcA-2           .LQQGGIKHSGIIAADFPGPGLIDSIIKLNGVHSNEKEILISQIPSESSPLSGQKNRSSQ
Bacillus_toyonensis_LY557_plcA-2     .LHQGEIKHVGIIAADFPGPGLIDSIIKLNGIHSNEKELLISQISSESSPLSGQQNRSSQ
Bacillus_cereus_LY460_plcA-2         .ILQGGIRHSGIIAADFPGPGLIDSIIKLNGINVNEKEILISQISSDSKPLPGEKNRSSQ
Listeria_monocytogenes_1AOD:A|PDBID  NLTSEKVRGLGILIMDFPEKQTIKNIIKNNKFN...........................


Bacillus_toyonensis_LY557_plcA-1

Bacillus_toyonensis_LY557_plcA-1     ............................................................
Bacillus_cereus_LY7_plcA-1           ............................................................
GCA_001044745.1_ASM104474v1_plcA-1   ............................................................
Bacillus_thuringiensis_LY178_plcA-1  ............................................................
Bacillus_thuringiensis_LY5_plcA-1    ............................................................
Bacillus_cereus_1GYM:A|PDBID|CHAIN|  ............................................................
Bacillus_thuringiensis_LY6_plcA-1    ............................................................
Bacillus_thuringiensis_LY1_plcA-1    ............................................................
GCA_001044955.1_ASM104495v1_plcA-1   ............................................................
GCA_001044645.1_ASM104464v1_plcA-1   ............................................................
GCA_001909175.1_ASM190917v1_plcA-1   ............................................................
Bacillus_cereus_LY2_plcA-1           ............................................................
Bacillus_cereus_LY460_plcA-1         ............................................................
Bacillus_cereus_LY14_plcA-1          ............................................................
GCA_001044475.1_ASM104447v1_plcA-1   ............................................................
GCA_001044735.1_ASM104473v1_plcA-1   ............................................................
GCA_002530625.1_ASM253062v1_plcA-1   ............................................................
GCA_002530635.1_ASM253063v1_plcA-1   ............................................................
GCA_002911655.1_ASM291165v1_plcA-1   ............................................................
GCA_003004435.1_ASM300443v1_plcA-1   ............................................................
GCA_002811445.1_ASM281144v1_plcA-1   ............................................................
GCA_003439755.1_ASM343975v1_plcA-1   ............................................................
GCA_001044555.1_ASM104455v1_plcA-1   ............................................................
Bacillus_thuringiensis_LY178_plcA-2  NFKIDSLPVGTKELKWVIEPSEKDYPYTISFNVMIDVSLGIDSTRWKNISHESRTEAYTN
Bacillus_thuringiensis_LY5_plcA-2    NFKIDSLPVGTKELKWVIEPSEKDYPSTISFNVMIDVSLGIDSTRWKNISHESRTEAYTN
Bacillus_thuringiensis_LY1_plcA-2    NFKIDSLPVGTKELKWVIEPSEKDYPSTISFNVMIDVSLGIDSTRWKNISHESRTEAYTN
Bacillus_cereus_LY2_plcA-2           NFKIDSLPVGTKELKWVIEPSEKDYPSTISFNVMIDVSLGIDSTRWKNISHGSRTEAYTN
Bacillus_cereus_LY7_plcA-2           NFKIDSLPVGTKELKWVIEPSEKDHPSTISFNVMIDVSLGIDSIRWKNISHGSRTEAYTN
Bacillus_toyonensis_LY557_plcA-2     NFRIDNLPVGTKELKWIIEPSEKDHPSTISFNVMIDVSLGTDSTRWKNISHGSRTEAYTN
Bacillus_cereus_LY460_plcA-2         NFSINNLPPGTIGLKWIIEPTQGETSDSISFNIMVDVSLGLDRVSWSNISNESRTAADTN
Listeria_monocytogenes_1AOD:A|PDBID  ............................................................
```

Bacillus_toyonensis_LY557_plcA-1

**Bacillus_toyonensis_LY557_plcA-1**	....
**Bacillus_cereus_LY7_plcA-1**	....
**GCA_001044745.1_ASM104474v1_plcA-1**	....
**Bacillus_thuringiensis_LY178_plcA-1**	....
**Bacillus_thuringiensis_LY5_plcA-1**	....
**Bacillus_cereus_1GYM:A|PDBID|CHAIN|**	....
**Bacillus_thuringiensis_LY6_plcA-1**	....
**Bacillus_thuringiensis_LY1_plcA-1**	....
**GCA_001044955.1_ASM104495v1_plcA-1**	....
**GCA_001044645.1_ASM104464v1_plcA-1**	....
**GCA_001909175.1_ASM190917v1_plcA-1**	....
**Bacillus_cereus_LY2_plcA-1**	....
**Bacillus_cereus_LY460_plcA-1**	....
**Bacillus_cereus_LY14_plcA-1**	....
**GCA_001044475.1_ASM104447v1_plcA-1**	....
**GCA_001044735.1_ASM104473v1_plcA-1**	....
**GCA_002530625.1_ASM253062v1_plcA-1**	....
**GCA_002530635.1_ASM253063v1_plcA-1**	....
**GCA_002911655.1_ASM291165v1_plcA-1**	....
**GCA_003004435.1_ASM300443v1_plcA-1**	....
**GCA_002811445.1_ASM281144v1_plcA-1**	....
**GCA_003439755.1_ASM343975v1_plcA-1**	....
**GCA_001044555.1_ASM104455v1_plcA-1**	....
**Bacillus_thuringiensis_LY178_plcA-2**	TKYYIASPIGATSKFTVKIYAITN
**Bacillus_thuringiensis_LY5_plcA-2**	TKYYIASPIGATRKFTVKIYAITN
**Bacillus_thuringiensis_LY1_plcA-2**	TKYYIASPIGATSKFTVKIYAITN
**Bacillus_cereus_LY2_plcA-2**	TKYYIASPIGATSKFTVKIYAITN
**Bacillus_cereus_LY7_plcA-2**	TKYYIASPIGATNKFTVKIYAITN
**Bacillus_toyonensis_LY557_plcA-2**	TKYYIASPIGATNKFTVKIYAITN
**Bacillus_cereus_LY460_plcA-2**	SKYYIANPSGVNHPFTVKVYAITN
**Listeria_monocytogenes_1AOD:A|PDBID**	....
